# Prevention of Typhoid by Vi Conjugate Vaccine and Achievable Improvements in Household Water, Sanitation, and Hygiene: Evidence From a Cluster-Randomized Trial in Dhaka, Bangladesh

**DOI:** 10.1093/cid/ciac289

**Published:** 2022-04-12

**Authors:** Birkneh Tilahun Tadesse, Farhana Khanam, Faisal Ahmmed, Justin Im, Md Taufiqul Islam, Deok Ryun Kim, Sophie S Y Kang, Xinxue Liu, Fahima Chowdhury, Tasnuva Ahmed, Asma Binte Aziz, Masuma Hoque, Juyeon Park, Gideok Pak, Khalequ Zaman, Ashraful Islam Khan, Andrew J Pollard, Jerome H Kim, Florian Marks, Firdausi Qadri, John D Clemens

**Affiliations:** International Vaccine Institute, Seoul, Republic of Korea; International Centre for Diarrhoeal Disease Research, Bangladesh, Dhaka, Bangladesh; International Centre for Diarrhoeal Disease Research, Bangladesh, Dhaka, Bangladesh; International Vaccine Institute, Seoul, Republic of Korea; International Centre for Diarrhoeal Disease Research, Bangladesh, Dhaka, Bangladesh; International Vaccine Institute, Seoul, Republic of Korea; International Vaccine Institute, Seoul, Republic of Korea; Oxford Vaccine Group, Department of Pediatrics, University of Oxford, and the National Institute for Health Research Oxford Biomedical Research Centre, Oxford, United Kingdom; International Centre for Diarrhoeal Disease Research, Bangladesh, Dhaka, Bangladesh; International Centre for Diarrhoeal Disease Research, Bangladesh, Dhaka, Bangladesh; International Vaccine Institute, Seoul, Republic of Korea; International Centre for Diarrhoeal Disease Research, Bangladesh, Dhaka, Bangladesh; International Vaccine Institute, Seoul, Republic of Korea; Cambridge Institute of Therapeutic Immunology and Infectious Disease, University of Cambridge School of Clinical Medicine, Cambridge Biomedical Campus, Cambridge, United Kingdom; International Vaccine Institute, Seoul, Republic of Korea; International Centre for Diarrhoeal Disease Research, Bangladesh, Dhaka, Bangladesh; International Centre for Diarrhoeal Disease Research, Bangladesh, Dhaka, Bangladesh; Oxford Vaccine Group, Department of Pediatrics, University of Oxford, and the National Institute for Health Research Oxford Biomedical Research Centre, Oxford, United Kingdom; International Vaccine Institute, Seoul, Republic of Korea; International Vaccine Institute, Seoul, Republic of Korea; Cambridge Institute of Therapeutic Immunology and Infectious Disease, University of Cambridge School of Clinical Medicine, Cambridge Biomedical Campus, Cambridge, United Kingdom; Department of Microbiology and Parasitology, University of Antananarivo, Antananarivo, Madagascar; Heidelberg Institute of Global Health, University of Heidelberg, Heidelberg, Germany; International Centre for Diarrhoeal Disease Research, Bangladesh, Dhaka, Bangladesh; International Vaccine Institute, Seoul, Republic of Korea; International Centre for Diarrhoeal Disease Research, Bangladesh, Dhaka, Bangladesh; Fielding School of Public Health, University of California, Los Angeles, Los Angeles, California, USA

**Keywords:** typhoid fever, WASH, typhoid conjugate vaccine, vaccine effectiveness

## Abstract

**Background:**

Typhoid fever contributes to approximately 135 000 deaths annually. Achievable improvements in household water, sanitation, and hygiene (WASH) combined with vaccination using typhoid conjugate vaccines (TCVs) may be an effective preventive strategy. However, little is known about how improved WASH and vaccination interact to lower the risk of typhoid.

**Methods:**

A total of 61 654 urban Bangladeshi children aged 9 months to <16 years, residing in 150 clusters with a baseline population of 205 760 residents, were randomized 1:1 by cluster to Vi-tetanus toxoid TCV or Japanese encephalitis (JE) vaccine. Surveillance for blood culture–confirmed typhoid fever was conducted over 2 years. Existing household WASH status was assessed at baseline as Better or Not Better using previously validated criteria. The reduction in typhoid risk among all residents associated with living in TCV clusters, Better WASH households, or both was evaluated using mixed-effects Poisson regression models.

**Results:**

The adjusted reduced risk of typhoid among all residents living in the clusters assigned to TCV was 55% (95% confidence interval [CI], 43%–65%; *P* < .001), and that of living in Better WASH households, regardless of cluster, was 37% (95% CI, 24%–48%; *P* < .001). The highest risk of typhoid was observed in persons living in households with Not Better WASH in the JE clusters. In comparison with these persons, those living in households with Better WASH in the TCV clusters had an adjusted reduced risk of 71% (95% CI, 59%–80%; *P* < .001).

**Conclusions:**

Implementation of TCV programs combined with achievable and culturally acceptable household WASH practices were independently associated with a significant reduction in typhoid risk.

**Clinical Trials Registration:**

ISRCTN11643110.

## Teaser

Better preexisting household water, sanitation, and hygiene and delivering Vi-TT vaccine act independently to reduce typhoid risk, so that the combination of both yields greater protection against typhoid at the population level (71%) than either vaccination (55%) or improved WASH alone (37%).

Typhoid fever is a systemic disease caused by *Salmonella enterica* serovar Typhi (*S*. Typhi) and is a major public health concern in low- and middle-income countries where the disease is endemic. Globally, an estimated 14 million persons develop typhoid fever and around 135 000 typhoid fever–related deaths occur annually, with the highest burden in children residing in urban slums [1, 2]. Emergence of multidrug-resistant *S*. Typhi further challenges conventional treatment regimens, resulting in a higher frequency of complications and adverse health outcomes [3, 4].

As a human-restricted pathogen, transmission of *S*. Typhi mainly occurs through ingestion of food or water contaminated with feces of infected persons. The risk of transmission of *S.* Typhi in people who have no access to safe drinking water is more than double that of those who have access to safe water [5]. Climate change and growth of economic opportunities in large cities has led to rapid urbanization of low- and middle-income countries; however, infrastructural development, such as waste management systems and provision of clean drinking water, has lagged behind. As a result, open sewage in urban slums often contaminates the domestic water source in urban dwellings, especially during the rainy season [6]. Furthermore, unhygienic practices during food handling exacerbate transmission within high-risk areas [7].

The combination of improvements in water, sanitation, and hygiene (WASH) and vaccination using safe and efficacious vaccines will be pivotal for typhoid fever prevention and potential elimination in endemic countries. However, establishment of municipal infrastructure for waste and sewage disposal and ensuring a sustainable uncontaminated water supply are constrained by the lack of financial capital and political will. As such, immunization with typhoid conjugate vaccines (TCVs), combined with building upon achievable and already existing improvements in household WASH, may be an effective strategy for typhoid prevention and potential elimination in hyperendemic urban slums [8–11]. However, little is known about how already existing improvements in household WASH and typhoid vaccination interact to lower the risk of typhoid. In a recent analysis, we demonstrated that salutary, already existing WASH practices and behaviors that are therefore feasible and affordable predicted a lower occurrence of typhoid fever in urban slums of Dhaka, Bangladesh (Tadesse BT, Khanam F, unpublished data).

Earlier, we reported results from a cluster-randomized trial in urban Bangladesh that demonstrated that administration of a single dose of Vi polysaccharide conjugated to tetanus toxoid (Vi-TT) TCV to children 9 months to <16 years of age conferred 85% protection against typhoid in these children over an 18-month period of surveillance [9]. Here, we extend these findings by addressing the public health question of what were the combined effects of Vi-TT vaccination of children and the level of preexisting household WASH in protecting the entire population of all ages against typhoid.

## METHODS

### Study Population

A cluster-randomized trial was conducted in a densely populated urban area of Mirpur, Dhaka, Bangladesh, that has endemic typhoid fever [9, 12]. A baseline census and biannual updates were conducted to enumerate the study population and to collect household-level demographic, socioeconomic, and WASH information. A total of 205 760 individuals were enumerated in the baseline census, and 61 654 children aged 9 months to <16 years were eligible for the vaccination at baseline. Information on births, deaths, and migrations that occurred in or out of the study area during the whole surveillance period were captured during biannual census updates.

### Vaccination

After the baseline census, the whole study area was divided into 150 geographical clusters, which were randomized at a 1:1 ratio to a single dose of either the intervention (Vi-TT, Typbar-TCV, Bharat Biotech International Ltd, Hyderabad, India) or control (SA 14-14-2 Japanese encephalitis [JE], Chengdu Institute of Biological Products, Chengdu, China) vaccine given to persons aged 9 months to <16 years. Neither the JE nor Vi-TT vaccines were available in the study area outside of the study setting. After the baseline immunization campaign, 3 catch-up vaccinations to the same age group were conducted at roughly 6-month intervals to maintain stable vaccine coverage considering the high migration rate within the study population. The baseline vaccination was held from 15 April to 15 May 2018 followed by catch-up vaccination campaigns from September to December 2018, April to May 2019, and October to November 2019.

### Typhoid Fever Surveillance

Eight health facilities located in and around the study area participated in passive surveillance serving the entire study population. These were all the public sector treatment centers for the catchment population, dispersed throughout the study area. All microbiology tests were done at a single reference laboratory at the International Centre for Diarrhoeal Disease Research, Bangladesh (icddr,b). Subjects were identified either by identification cards distributed during the census or, lacking such cards, with use of the computerized census using tablets at each surveillance site. Passive surveillance for typhoid fever was initiated on 26 February 2018. Individuals with fever, defined as a history of fever for ≥2 days or axillary temperature of ≥38°C, were enrolled after giving informed consent. Blood samples were collected from study participants and assessed by microbiological cultures for bacterial growth. Febrile visits were concatenated into febrile episodes when initial and subsequent visits occurred within 14 days of one another. An episode of typhoid fever was defined as a febrile episode in which *S*. Typhi was isolated from blood culture and in which a home visit confirmed that the person whose name was given at the treatment center had indeed sought care for treatment of fever on the date of presentation.

### Definition of Household WASH

In a recent analysis (Tadesse BT, Khanam F, unpublished data), we developed a composite dichotomous variable for household WASH (Better, Not Better) using individual WASH variables collected during the baseline census for those present in the study area during the baseline census, and during the first census update after the onset of their first participation for those who moved into the clusters after the baseline census and for births. A household with Better WASH had characteristics of owning a private toilet and the availability of a water filter in the household during visit by study staff; or owning a private toilet and access to safe drinking water, defined as having a private tap, private well, bottled water, or water vendor (Supplementary Figure 1).

### Statistical Analysis

The analysis was conducted in a dynamic population that considered births and in-migrations as well as catch-up vaccination after the baseline vaccination. Follow-up began at the “start date of residence.” For those present at baseline the start date of residence was the date of vaccination for vaccinees and the midpoint of the baseline vaccination (30 April 2018) for nonvaccinees. For those who in-migrated or were newly born after baseline, the date of first in-migration and the date of birth were considered as the start date of residence in the study area. Each individual was analyzed following the “first in, first out” principle where individuals were included at the time of the start date of residence in the study cluster and were censored at death, end of surveillance (15 March 2021), or migration out of the cluster, whichever came first.

We evaluated the impact of vaccination with Vi-TT by comparing the rate of blood culture–confirmed typhoid fever episodes in the overall population residing in the Vi-TT clusters with those living in the JE clusters. Only first typhoid episodes occurring 1 or more days after the start date of residence were included in the analysis. The association between living in a Better WASH household and typhoid was assessed using the same start date and follow-up strategy. We then evaluated the overall protection of all individuals living in Vi-TT vs JE clusters according to whether they lived in Better vs Not Better WASH households, and the protection of living in Better vs Not Better WASH households by their residence in Vi-TT vs JE clusters. Finally, we evaluated the evidence for a trend of the incidence of typhoid considering 4 groups: persons living in Not Better WASH households in JE clusters (the reference group), persons living in Better WASH households in JE clusters, persons living in Not Better WASH households in Vi-TT clusters, and persons living in Better WASH households in Vi-TT clusters.

In simple analyses, Kaplan-Meier survival curves were constructed to evaluate protection against typhoid fever associated with residence in a Vi-TT cluster or with residence in a household with Better WASH, statistically assessed using the log-rank test [13]. We further applied mixed-effects Poisson regression models to estimate the incidence rate ratios (IRRs) related to residence in a Vi-TT cluster or in a household with Better WASH while adjusting for the design effect of cluster-randomization including those used to define the strata for randomization (geographical ward, distance to study clinics), the number of eligible children at baseline, and other baseline prespecified covariates during protocol development, including age at baseline census and sex [14]. The IRRs and their *P* values and 95% confidence intervals (CIs) were estimated by exponentiating the coefficients for dichotomous variables for the vaccine arm of residence and for residence in a Better vs Not Better WASH household, and protective effectiveness (PE) associated with residence in a Vi-TT cluster and in a Better WASH household was calculated as (1 – IRR) × 100%. An interaction term for cluster assignment and household WASH was fitted in the models to evaluate the possibility of synergistic protection against typhoid between living in Vi-TT clusters and living in a household with Better WASH. A 2-tailed *P* value < .05 was considered as the threshold for significance. All analyses were carried out using R version 4.1.0 and the glmmTMB package was used to fit the mixed-effects Poisson regression models [15, 16].

## RESULTS

### Trial Participants

**Figure 1.**
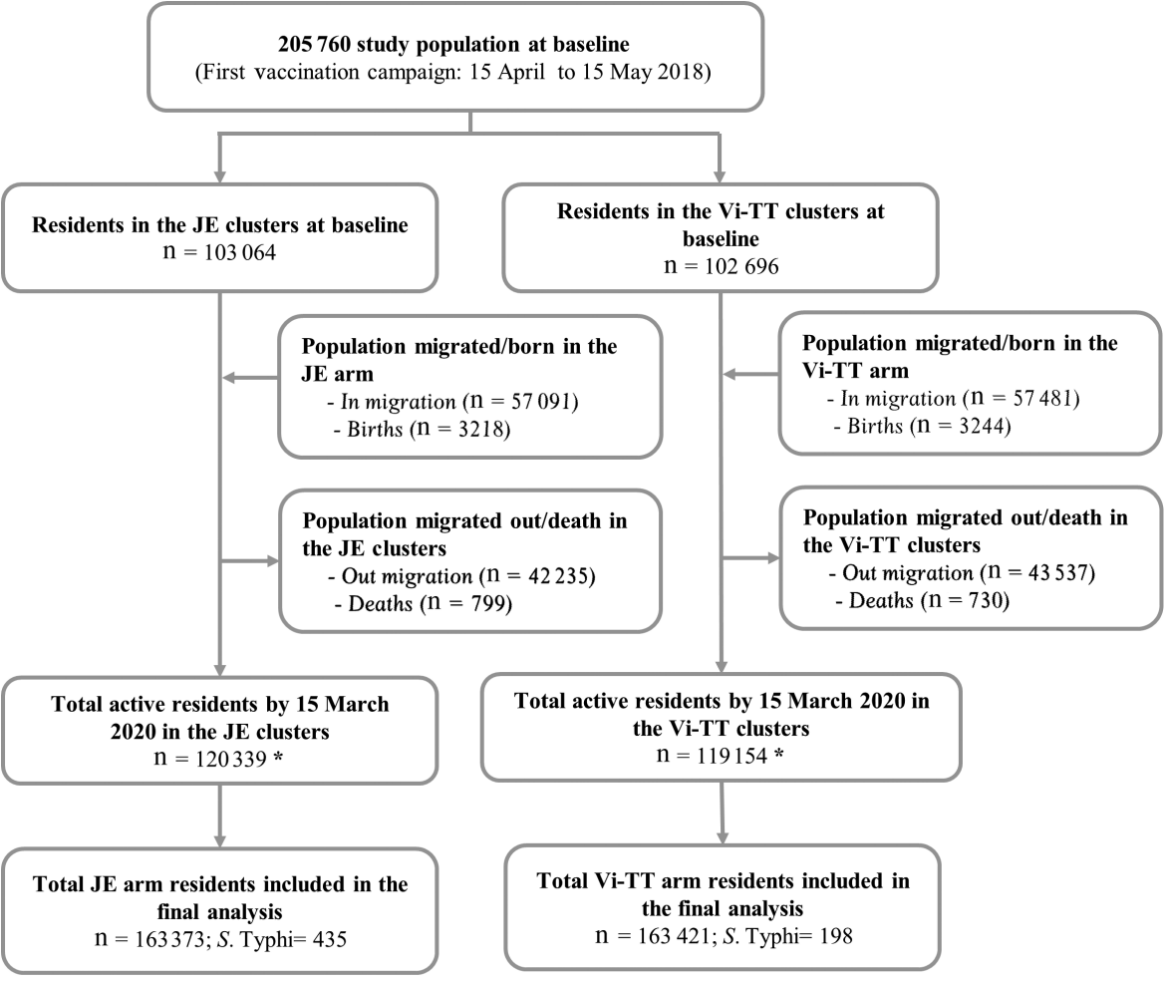
Consolidated Standards of Reporting Trials (CONSORT) diagram showing population at first and last census and incident typhoid cases in Vi polysaccharide conjugated to tetanus toxoid (Vi-TT) and Japanese encephalitis (JE) clusters. *Three study participants (1 from the JE and 2 from the Vi-TT clusters) died by 15 March 2020.

The disposition of trial participants using the Consolidated Standards of Reporting Trials (CONSORT) diagram is presented in Figure 1. In total, 205 760 individuals lived in the study area during the baseline census; of these, 102 696 and 103 064 resided in the Vi-TT and JE clusters, respectively. During 24 months of follow-up, we recorded 94 572 in-migrations and 6462 births and 85 772 out-migrations and 1529 deaths. The total population residing in the study area was 239 493 at the last census and cumulatively 326 794 at any time during the entire 24 months of follow-up. A total of 33 727 and 33 315 individuals received a single dose of Vi-TT and JE vaccine, respectively. The mean age (± standard deviation) of the residents at start date of residence was 25.3 (±17.2) years in the Vi-TT clusters and 25.4 (±17.3) years in the JE clusters. As shown in Table 1, the populations residing in the Vi-TT and JE clusters were also comparable at the start date of residence for distribution by sex, religion, ward of residence, household WASH status, and prevalence of Better WASH household status (35% in the Vi-TT clusters and 33% in the JE clusters) (Table 1). The distribution of baseline characteristics by residence in Better and Not WASH household is presented in Supplementary Table 1.

**Table 1.** Baseline Demographic Characteristics of Study Participants Living in the Japanese Encephalitis and Vi–Tetanus Toxoid Clusters at Baseline Census

Parameter	JE Group	Vi-TT Group
All residents	163 373	163 421
Mean age ± SD^a^	25.4 ± 17.3	25.3 ± 17.2
Sex		
Male	81 134 (49.7)	81 097 (49.6)
Female	82 239 (50.3)	82 324 (50.4)
Religion		
Muslim	161 591 (98.9)	161 150 (98.6)
Others	1782 (1.1)	2271 (1.4)
Ward of residence		
2	62 791 (38.4)	66 077 (40.4)
3	38 551 (23.6)	44 168 (27.0)
5	62 031 (38.0)	53 176 (32.5)
Resident’s household WASH status		
Better	56 969 (34.9)	54 281 (33.2)
Not better	106 404 (65.1)	109 140 (66.8)
Better WASH coverage, %, Mean ± SD	37.6 ± 16.5	35.7 ± 18.3

Abbreviations: JE, Japanese encephalitis vaccine; SD, standard deviation; Vi-TT, Vi polysaccharide conjugated to tetanus toxoid; WASH, water, sanitation, and hygiene.

a Age in years measured at baseline census.

### Protection Against Typhoid Associated With Living in Vi-TT Clusters and Better WASH Households

In the cumulative population ever followed in the clusters, vaccine coverage was 21% in the Vi-TT clusters and 20% in the JE clusters; in both Vi-TT and JE clusters, vaccine coverage of the age-eligible population was maintained at around 64%. A total of 633 confirmed typhoid fever cases were diagnosed in the study population during the approximately 24 months of follow-up. Of these, 435 cases occurred in the JE clusters and 198 in the Vi-TT clusters with respective typhoid fever incidence rates of 209 and 96 per 100 000 person-years of observation (PYO). The adjusted overall PE associated with living in the Vi-TT clusters was 55% (95% CI, 43%–65%; *P* < .001). Similarly, 159 cases of typhoid fever were diagnosed in persons living in Better WASH households compared with 474 cases in those living in Not Better WASH households with respective incidence rates of 103 and 181 per 100 000 PYO. The PE associated with living in Better WASH households was 37% (95% CI, 24%–48%; *P* < .001; Table 2).

**Table 2.** Protection Against Typhoid Associated With Residence in a Vi–Tetanus Toxoid Cluster and Residence in a Better Water, Sanitation, and Hygiene Household

Vaccine/WASH	No. of Individuals	No. of Cases	PY	IR^a^	Crude PE, % (95% CI)	*P* Value	Adjusted PE^a^, % (95% CI)	*P* Value
JE	163 373	435	208 414	209	Ref	…	Ref	…
Vi-TT	163 421	198	206 867	96	56 (43–66)	<.001	55 (43–65)	<.001
Not Better	215 544	474	261 425	181	Ref	…	Ref	…
Better	111 250	159	153 856	103	42 (30–52)	<.001	37 (24–48)	<.001

Abbreviations: CI, confidence interval; IR, incidence rate; JE, Japanese encephalitis vaccine; PE, protective effectiveness; PY, person-years; Ref, reference group; Vi-TT, Vi polysaccharide vaccine conjugated with tetanus toxoid; WASH, water, sanitation, and hygiene.

a Model adjusted for cluster-randomization variables (ward [ward 2, ward 3, ward 5], number of eligible children at baseline, design effect, age at baseline census, and sex).

We next evaluated the overall PE associated with living in Vi-TT clusters stratified by household WASH status, and with living in Better WASH households stratified by vaccine cluster arm assignment. For persons living in Not Better WASH households, the adjusted overall PE associated with living in Vi-TT clusters was 58% (95% CI, 45%–69%; *P* < .001) while for those living in Better WASH households, the adjusted PE was 48% (95% CI, 26%–64%; *P* < .001). Similarly, living in Better WASH households was associated with a PE of 39% (95% CI, 24%–51%; *P* < .001) in persons living in the JE clusters compared to 34% (95% CI, 7%–53%; *P* < .001) in those living in the Vi-TT clusters (Table 3).

**Table 3.** Protection Against Typhoid Associated With Residence in a Vi-TT Cluster, Stratified by Household Water, Sanitation, and Hygiene (WASH), and With Residence in a Household With Better WASH, Stratified by Vaccination Cluster

Strata	Group	No. of Individuals	No. of Cases	PY	IR	Crude PE, % (95% CI)	*P* Value	Adjusted PE^a^, % (95% CI)	*P* Value
Not Better WASH	JE	106 404	325	129 088	252	Ref	…	Ref	…
	Vi-TT	109 140	149	132 337	113	58 (44–69)	<.001	58 (45–69)	<.001
Better WASH	JE	56 969	110	79 326	139	Ref	…	Ref	…
	Vi-TT	54 281	49	74 530	66	52 (30–67)	<.001	48 (26–64)	<.001
JE Clusters	Not Better WASH	106 404	325	129 088	252	Ref	…	Ref	…
	Better WASH	56 969	110	79 326	139	44 (30–55)	<.001	39 (24–51)	<.001
Vi-TT Clusters	Not Better WASH	109 140	149	132 337	113	Ref	…	Ref	…
	Better WASH	54 281	49	74 530	66	40 (16–57)	.003	34 (7–53)	.016

Abbreviations: CI, confidence interval; IR, incidence rate; JE, Japanese encephalitis vaccine; PE, protective effectiveness; PY, person-years; Ref, reference group; Vi-TT, Vi polysaccharide vaccine conjugated with tetanus toxoid; WASH, water, sanitation, and hygiene.

a Model adjusted for cluster-randomization variables (ward [ward 2, ward 3, ward 5], number of eligible children at baseline, design effect, age at baseline census, and sex).

### Combined Impact of Living in Better WASH Households and Within Vi-TT Clusters

We further analyzed the combined impact of living in Better WASH households within the Vi-TT clusters by examining 4 groups defined by living in Vi-TT vs JE clusters, and by living in households with Better vs Not Better WASH. We observed the lowest typhoid incidence in persons who lived in Better WASH households located within the Vi-TT clusters, with an incidence rate of 66 per 100 000 PYO and an adjusted PE of 71% (95% CI, 59%–80%; *P* < .001) in comparison with the population living in Not Better WASH households in the JE clusters (Table 4). This PE was higher than that observed for living in Vi-TT clusters but within Not Better WASH households, with an adjusted PE of 57% (95% CI, 44%–67%; *P* < .001), and those living in Better WASH households but within the JE clusters, with an adjusted PE of 39% (95% CI, 24%–51%, *P* < .001) (Table 4). The proportion of vaccinees in these 4 categories was comparable (18%–21%). No evidence of statistical interaction between household WASH status and cluster assignment was observed (*P* = .740).

**Table 4.** Protection Against Typhoid by Vaccine Cluster of Residence and Water, Sanitation, and Hygiene in the Household

Vaccine + WASH	No. of Individuals	No. of Cases	PY	IR	Crude PE, % (95% CI)	*P* Value	Adjusted PE^a^, % (95% CI)	*P* Value
JE + Not Better WASH	106 404	325	129 088	252	Ref	…	Ref	…
JE + Better WASH	56 969	110	79 326	139	44 (30–55)	<.001	39 (24–51)	<.001
Vi-TT + Not Better WASH	109 140	149	132 337	113	57 (43–67)	<.001	57 (44–67)	<.001
Vi-TT + Better WASH	54 281	49	74 530	66	74 (63–82)	<.001	71 (59–80)	<.001

Abbreviations: CI, confidence interval; IR, incidence rate; JE, Japanese encephalitis vaccine; PE, protective effectiveness; PY, person-years; Ref, reference group; Vi-TT, Vi polysaccharide vaccine conjugated with tetanus toxoid; WASH, water, sanitation, and hygiene.

a Model adjusted for cluster-randomization variables (ward [ward 2, ward 3, ward 5], number of eligible children at baseline, design effect, age at baseline census, and sex).

## DISCUSSION

Our findings show that already existing and therefore culturally acceptable and achievable improvements to household WASH practices and behaviors, combined with vaccination with an efficacious typhoid vaccine, were associated with a significant reduction in the burden of typhoid fever in an endemic urban slum in Bangladesh. Living in a cluster receiving Vi-TT (55% overall protection) or residing in a household with Better WASH (37% overall protection) were each independently associated with a lower risk of typhoid. Moreover, examination of the combination of residence in a Better WASH household and residence in a cluster receiving Vi-TT revealed a stepwise increase in protection, from living in a Better WASH household in a JE cluster (39%), to living in a Vi-TT cluster but in a Not Better WASH household (57%), to living in a Better WASH household within a Vi-TT cluster (71%).

Our study had several potential limitations. Households were not randomized to Better vs Not Better WASH. However, controlling for confounding variables did not affect our results. Furthermore, the composite household WASH variable was built based on a simplified evaluation of household-level WASH features ascertained via a single questionnaire at baseline. This could have led to loss of some information, which might have led to underestimation of the actual impact of Better WASH household status. A more comprehensive and specific definition of WASH behaviors and facilities responsible for reducing typhoid transmission would have led to higher estimates of protection. Additionally, the clusters were not true epidemiological units of person-to-person typhoid transmission and likely experienced a great deal of transmission originating from the outside, which would have attenuated estimates of overall Vi-TT protection [9]. The relatively low sensitivity of blood culture for the detection of *S.* Typhi could also have led to underestimation of the absolute effect size of the interventions, though because differential sensitivity of blood cultures by vaccine or WASH status would seem unlikely, the relative reductions of typhoid rates reported should not have been affected. Finally, it is also important to stress that the WASH features studied in this analysis represent existing WASH behaviors and practices of this population. Whether WASH interventions delivered to at-risk populations can replicate or even improve upon these behaviors and practices in entire populations represents a significant challenge for the future.

Despite these limitations, our analysis had several strengths. First, it was based on a rigorously planned and implemented cluster-randomized trial in which vaccine assignment was unambiguous. In this trial, the diagnosis of typhoid was made blinded to vaccine assignment. As well, persons conducting surveillance of typhoid had no foreknowledge of our hypothesis regarding the relationship between WASH and typhoid. Second, we used a WASH composite variable that was previously developed in the nonintervention clusters in the same community and was validated in a population separate from the population in which the composite variable was derived.

Our findings support a concerted approach to typhoid elimination through vaccination using safe and effective TCVs with implementation of achievable WASH improvements in high-risk communities. It is, however, important to note that the combination of vaccination using TCVs and living in a Better WASH household still reflected only a 70% reduction in relation to the absence of both, though, as noted earlier, our estimates of the combined effect of vaccination and WASH were likely conservative for methodological reasons. We therefore suggest that this reduction probably represents the minimum that can be achieved in realistic programs of vaccination and effective and feasible WASH adaptations. Future well-designed studies of WASH interventions and vaccination will be needed to design effective elimination strategies.

Taken together, our findings help elucidate the separate and combined impact of vaccination with TCV and already existing improvements in WASH practices and behaviors in preventing typhoid in hyperendemic urban slums. These findings can be taken as a “proof of principle” to support the assertion that implementation of achievable, high-impact WASH interventions that leverage already existing salutary practices, in combination with vaccination programs, may control typhoid fever in such settings.

## Supplementary Data

Supplementary materials are available at *Clinical Infectious Diseases* online. Consisting of data provided by the authors to benefit the reader, the posted materials are not copyedited and are the sole responsibility of the authors, so questions or comments should be addressed to the corresponding author.

## Notes

***Financial support.*** This work was supported by the Bill & Melinda Gates Foundation (INV-025386). A. P. reports funding from the UK National Institute for Health Research.

## Supplementary Material

ciac289_Supplementary_Data
