# Supplementary material for: Prevention of Typhoid by Vi Conjugate Vaccine and Achievable Improvements in Household Water, Sanitation, and Hygiene: Evidence From a Cluster-Randomized Trial in Dhaka, Bangladesh
Source: Clin Infect Dis. 2022 Apr 12;75(10):1681–7. doi: 10.1093/cid/ciac289 (PMC9662172; doi:10.1093/cid/ciac289)
Supplement: ciac289_Supplementary_Data [file ciac289_supplementary_data.zip › Supplementary Table 1.docx]

**Supplementary Table 1**: Baseline demographic characteristics of study participants living in the not better and better wash HH at baseline census.

| **Parameter** | **Not-better wash HH** | **Better wash HH** |
| --- | --- | --- |
| All residents | 215544 | 111250 |
| Average Age ± SD* | 24.5 ± 16.6 | 27.0 ± 18.3 |
| Sex |  |  |
| Male | 106592 (49.5) | 55611 (50.0) |
| Female | 108952 (50.5) | 55611 (50.0) |
| Religion |  |  |
| Muslim | 212986 (98.8) | 109755 (98.7) |
| Others | 2558 (1.2) | 1495 (1.3) |
| Ward of residence |  |  |
| 2 | 79699 (37.0) | 49169 (44.2) |
| 3 | 50316 (23.3) | 32403 (29.1) |
| 5 | 85529 (39.7) | 29678 (26.7) |
| Study Arm |  |  |
| JE Vaccine | 106404 (49.4) | 56969 (51.2) |
| Vi-TT Vaccine | 109140 (50.6) | 54281 (48.8) |
| Better WASH coverage (%) ± SD | 32.2 ± 16.4 | 45.4 ± 16.2 |

^*^Age in years measured at baseline census; SD – Standard Deviation; JE – Japanese Encephalitis; Vi-TT – Vi polysaccharide vaccine conjugated to tetanus toxoid; HH – Household; WASH – Water, Sanitation and Hygiene
