# Supplementary figures and images for: Prevention of Typhoid by Vi Conjugate Vaccine and Achievable Improvements in Household Water, Sanitation, and Hygiene: Evidence From a Cluster-Randomized Trial in Dhaka, Bangladesh
Source: Clin Infect Dis. 2022 Apr 12;75(10):1681–7. doi: 10.1093/cid/ciac289 (PMC9662172; doi:10.1093/cid/ciac289)

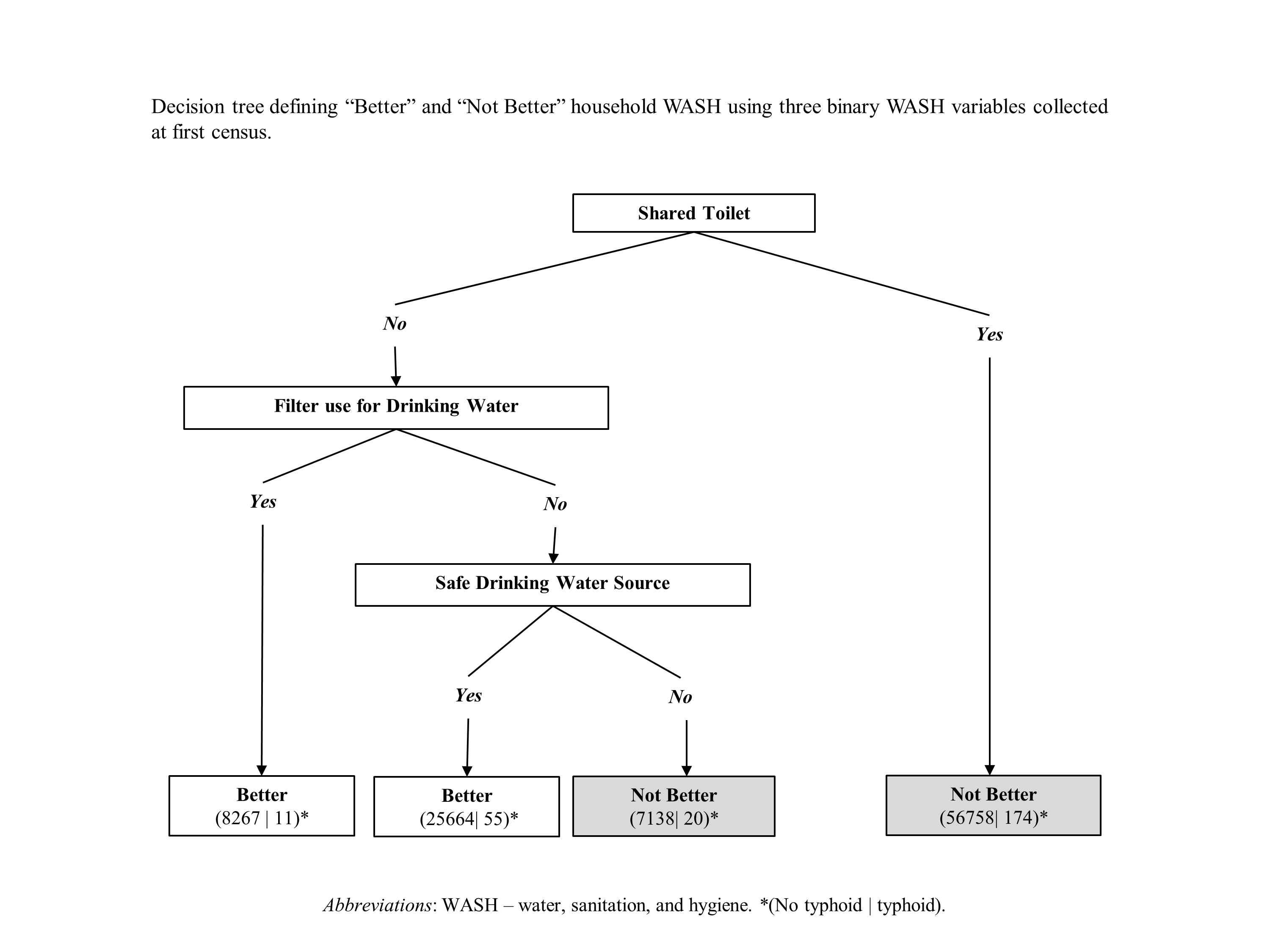

Supplement: ciac289_Supplementary_Data [file ciac289_supplementary_data.zip › Supplementary Figure 1.tif]
